# A nontraditional presentation and treatment for emphysematous cystitis

**DOI:** 10.1016/j.eucr.2023.102321

**Published:** 2023-01-11

**Authors:** Benjamin Becker, Asher George, Ryan Owen

**Affiliations:** a Texas Tech University Health Sciences Center School of Medicine, 3601 4th St, Lubbock, TX, 79430, USA; b Covenant Medical Center, 3615 19th St, Lubbock, TX, 79410, USA

**Keywords:** Cystitis, Emphysematous, Rupture, Management

## Abstract

Emphysematous cystitis (EC) is a bladder pathology typically resulting from gas-producing bacterial infections. Risk factors include female gender, age greater than 60, diabetes mellitus, glycosuria, and urinary stasis. If not addressed promptly, it can progress to critical conditions. Management depends on severity, ranging from conservative to surgical interventions. We present a unique case of EC in a patient who lacked the most common risk factors, and who experienced a spontaneous intraperitoneal bladder rupture. Additionally, we treated this presentation against what historically is suggested. Lastly, this is the first published incidence of a patient with both Parkinson's Disease and EC.

## 1 Introduction

Emphysematous cystitis (EC) is a life-threatening bladder condition resulting from gas-producing bacterial infections*.*^1^ Some of the risk factors include female gender, age greater than 60-years-old, diabetes mellitus (DM), and urinary stasis. Complications of EC include bladder rupture, peritonitis, and/or emphysematous pyelonephritis. Management depends on severity, ranging from bladder drainage and antibiotics, to cystectomy or other surgical interventions.

We report our experience with a patient who presented with EC and lacked major risk factors. This presentation was also complicated by an intraperitoneal (IP) bladder wall rupture, which also typically occur due to a different mechanism. Treatment was conservative given our patient's high risk for complications, even though that traditional methods involve surgery and that the peritoneum had already been exposed. Additionally, to our knowledge, this is the first incidence in literature of a patient with both Parkinson's Disease (PD) and EC.

## 2 Case presentation

**Fig. 1:**
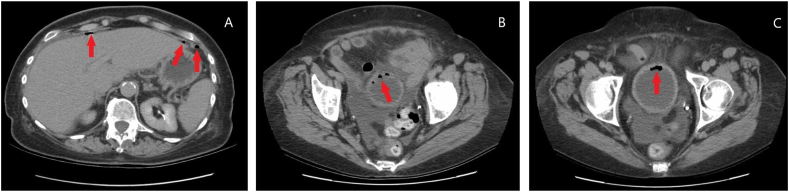
CT A/P with IV contrast. A: free air anterior to liver and stomach (denoted by red arrows). B (more superior): free air within the antero-superior aspect of the bladder wall (denoted by red arrow). C (more inferior): free air in the anterior bladder wall and lumen (denoted by red arrow), as well as free peritoneal air anterior to the bladder. (For interpretation of the references to colour in this figure legend, the reader is referred to the Web version of this article.)

Our patient is a 78-year-old female with a past medical history of PD who presented to the emergency center with a chief complaint of acute, dull, and diffuse abdominal pain. She was also experiencing incontinence, constipation, fever, and chills. Computed tomography scan of the abdomen and pelvis (CT A/P) with intravenous (IV) contrast revealed free air within the abdomen located anteriorly to the liver and adjacently to the stomach and splenic flexure, a large amount of free abdominal fluid, and trace air within the bladder (Fig. 1). It was deemed necessary to perform an exploratory laparotomy.

A large volume of purulent fluid was exposed upon entering the peritoneum, which was subsequently drained and sent for cultures. Furthermore, a complete bowel assessment revealed no evidence of perforation. Thickened areas of vaginal cuff and bladder tissue were observed intraoperatively, but no fistulous connection or perforation was present.

Examination of the urinary system revealed a necrotic and excoriated bladder consisting of a defect in the bladder dome, prompting an immediate frozen-section biopsy. This yielded benign fibrogranulation tissue with chronic inflammation, ruling out a malignancy. The bladder wall was friable and thickened causing poor compliance. Due to the appearance of the bladder intraoperatively, we favored resection rather than closure. The operation proceeded with a multi-layered cystorrhaphy and a mobilized omental flap, and was concluded with placement of a closed-suction abdominal drain and Foley catheter. Broad spectrum antibiotics were started immediately post-operatively.

Initial recovery period was complicated by leukocytosis and monitored until time of discharge, but otherwise was uneventful. Days after operation, intraoperative fluid cultures returned with growth only for *Escherichia coli* (*E. coli*) with extended-spectrum beta-lactamases (ESBL) sensitivity, to which we immediately narrowed antibiotic treatment to IV meropenem. Patient was discharged on post-operative day 12, and followed-up in clinic on multiple occasions with normal bladder function.

The final diagnoses in our patient include EC secondary to an *E. coli* UTI and peritonitis secondary to IP bladder perforation.

## 3 Discussion

EC is a rare condition featuring air confined to the bladder wall and lumen. It can present asymptomatically, with minimal pneumaturia or voiding symptoms, or even with acute abdomen and sepsis.^1^ *E. coli* is the most common culprit, found in over half of all cases. Patients with DM and who were female, the two greatest risk factors, are twice as likely to develop this condition. However, our patient did not have a history of DM, and rather her presentation was most likely due to progression of a UTI secondary to urinary outflow obstruction.

General management of EC includes bladder drainage, glycemic control, and antibiotics.^1^ Those that fail to respond or progress to life-threatening complications, such as IP bladder wall rupture, typically require immediate surgical repair.

IP bladder injuries are less common than extraperitoneal (EP), and they usually occur secondary to high impact injuries and in weak areas, such as the bladder dome.^2^ Rather than abdominal trauma, the bladder dome rupture in our patient occurred due to overextension by gas-forming bacteria.

IP bladder injuries can have life-threatening consequences, and thus treatment should begin promptly. Both surgical and non-surgical approaches have been reported. Historically, IP bladder injuries were repaired with surgical approach, such as cystectomy, because it was thought that IP rupture had low rates of spontaneous healing and high risk of complications.^3^Additionally, surgery may be required in the 10% of patients who do not respond to non-surgical management and/or develop severe infection. However, non-operative treatment with antibiotics and urinary catheterization have become the current preferred method, due to decreased risk for infections, reduced bleeding, and shorter healing times.^3^ It could be posed that we should have surgically managed this rupture, especially since we had already entered the peritoneum while performing diagnostic laparoscopy. However, we elected to treat conservatively because our patient's age, frailty, and comorbid condition put her at high risk for complications.

PD increases a patient's risk for complications post-operatively, such as pulmonary embolism, aspiration pneumonia, UTI, and acute renal failure.^4^ This also supports our decision to treat our patient conservatively rather than with more operations. While it is known that neurogenic bladder can occur secondary to PD (and thus, a possible contributor to EC development),^5^ to our knowledge, this is the first incidence of EC in a patient with PD in literature. Our goal with this case report is to provide outcomes-based guidance for clinicians that encounter this rare presentation of EC.

Our patient has not experienced any complications during the follow-up period thus far. She has demonstrated low post-void residuals at each clinic visit, an outcome which would not have been achievable if we performed additional surgery. Further research should be performed to determine if a relationship exists between EC and PD.

## 4 Conclusion

EC is essential to diagnose and recognize, since it can quickly progress to life-threatening conditions. This report highlights that EC can present in an atypical fashion, and that treatment should account for patient-specific factors, even if it deviates from the norm.

## Funding

This research did not receive any specific grant from funding agencies in the public, commercial, or not-for-profit sectors.

## Author contributions

Benjamin Becker: Writing- Original draft preparation, Visualization. Asher George: Writing- Original draft preparation. Ryan Owen: Writing- Reviewing and Editing, Supervision.

## Declaration of competing interest

None.
